# OP19 Clinical Effectiveness Of Glucocorticoids For The Treatment Of Knee Osteoarthritis – Systematic Review And Longitudinal Meta-Analysis

**DOI:** 10.1017/S0266462325100779

**Published:** 2025-12-29

**Authors:** Magdalena Moshi, Alvin Atlas, John Rathbone, Danielle Stringer, Thomas Vreugdenburg

## Abstract

**Introduction:**

Globally, knee osteoarthritis (OA) is a prevalent and disabling condition. Intra-articular glucocorticoid injection (IAGI) is widely used to treat knee OA. However, there is conflicting evidence on the benefit-harm profile of IAGI for this indication. This study aimed to determine the clinical effectiveness and safety of IAGI for knee OA.

**Methods:**

Systematic literature searches were conducted in three biomedical databases (MEDLINE, Embase, the Cochrane Library) through to 4 October 2023. Randomized controlled trials (RCTs) that compared IAGI with no treatment, sham injection, or oral placebo in patients with knee OA were included. RCTs were appraised using the revised Cochrane risk-of-bias tool. Longitudinal meta-analysis (LMA) was conducted for predetermined outcomes (i.e., pain, function, health-related quality of life [HRQoL], care utilization, adverse events [AEs], and serious adverse events) at one month, three months, six months, and 12 months. Pairwise meta-analyses were conducted at individual time points if there was insufficient evidence to conduct a LMA. The research project was funded by the Swiss Federal Office of Public Health.

**Results:**

In total, 16 RCTs comprising 1,522 patients with knee OA were included. The LMA reported a small reduction in pain at one month (standardized mean difference: −0.30, 95% confidence interval [CI]: −0.52, −0.08), but no clinically or statistically significant differences from three to 12 months. Regarding function, the LMA found no significant differences at any time point. Pairwise meta-analyses at one month showed reduced care utilization favoring IAGI (mean difference −0.43; 95% CI: −0.81, −0.05), but not at other time points, and no differences in HRQoL at any time point. No significant safety concerns were found in relation to AEs and serious AEs.

**Conclusions:**

IAGI provided short-term pain relief for knee OA at one month post-injection, with beneficial effects diminishing by three months and beyond. There were no substantial improvements in function or HRQoL. No significant safety concerns were identified. To guide clinical decision-making, further research should focus on HRQoL as well as potential subgroups of patients that may benefit in the longer term.

